# Supplementary material for: Self-regulated learning strategies and academic achievement in South Korean 6th-graders: A two-level hierarchical linear modeling analysis
Source: PLoS One. 2023 Apr 26;18(4):e0284385. doi: 10.1371/journal.pone.0284385 (PMC10132645; doi:10.1371/journal.pone.0284385)
Supplement: S1 Appendix — (DOCX) [file pone.0284385.s001.docx]

**Appendix. HLM Equation**

**CS Use on Literacy Achievement (Final Model in Table 3):**

*LITERACY_ACHEIVEMENT_ij_* = *γ_00_* + *γ_01_***SCHTYPE_j_* + *γ_02_***REHEARSAL_j_* + *γ_03_***ELABORATION_j_* 
     + *γ_04_***ORGANIZATION_j_* + *γ_05_***METACOGNITION_j_* + *γ_06_***URBAN_j_* + *γ_07_***SUBURBAN_j_* 
    + *γ_10_***GENDER_ij_* + *γ_11_***SCHTYPE_j_***GENDER_ij_* + *γ_12_***URBAN_j_***GENDER_ij_* + *γ_13_***SUBURBAN_j_***GENDER_ij_* 
    + *γ_20_***REHEARSAL_ij_* 
    + *γ_30_***ELABORATION_ij_* 
    + *γ_40_***ORGANIZATION_ij_* + *γ_41_***SCHTYPE_j_***ORGANIZATION_ij_* + *γ_42_***URBAN_j_***ORGANIZATION_ij_* + *γ_43_***SUBURBAN_j_***ORGANIZATION_ij_* 
    + *γ_50_***METACOGNITION_ij_* 
    + *u_0j_* + *u_1j_***GENDER_ij_* 
    + *u_4j_***ORGANIZATION_ij_* + *r_ij_*

**CS Use on Math Achievement (Final Model in Table 3):**

*MATH_ACHEIVEMENT_ij_* = *γ_00_* + *γ_01_***SCHTYPE_j_* + *γ_02_***REHEARSAL_j_* + *γ_03_***ELABORATION_j_* 
    + *γ_04_***ORGANIZATION_j_* + *γ_05_***METACOGNITION_j_* + *γ_06_***URBAN_j_* + *γ_07_***SUBURBAN_j_* 
    + *γ_10_***GENDER_ij_* 
    + *γ_20_***REHEARSAL_ij_* 
    + *γ_30_***ELABORATION_ij_* 
    + *γ_40_***ORGANIZATION_ij_* 
    + *γ_50_***METACOGNITION_ij_* + *γ_51_***SCHTYPE_j_***METACOGNITION_ij_* + *γ_52_***URBAN_j_***METACOGNITION_ij_* + *γ_53_***SUBURBAN_j_***METACOGNITION_ij_* 
    + *u_0j_* + *u_5j_***METACOGNITION_ij_* + *r_ij_*

**BS Use on Literacy Achievement (Final Model in Table 4):**

*LITERACY_ACHEIVEMENT_ij_* = *γ_00_* + *γ_01_***SCHTYPE_j_* + *γ_02_***TASK_REGULATION_j_* + *γ_03_***EFFORT_REGULATION_j_* 
    + *γ_04_***TIME_REGULATION_j_* + *γ_05_***ENVIRONMENTAL_REGULATION_j_* + *γ_06_***HELP_SEEKING_j_* + *γ_07_***URBAN_j_* 
    + *γ_08_***SUBURBAN_j_* 
    + *γ_10_***GENDER_ij_* 
    + *γ_20_***TASK_REGULATION_ij_* 
    + *γ_30_***EFFORT_REGULATION_ij_* 
    + *γ_40_***TIME_REGULATION_ij_* 
    + *γ_50_***ENVIRONMENTAL_REGULATION_ij_* + *γ_51_***SCHTYPE_j_***ENVIRONMENTAL_REGULATION_ij_* + *γ_52_***URBAN_j_***ENVIRONMENTAL_REGULATION_ij_* + *γ_53_***SUBURBAN_j_***ENVIRONMENTAL_REGULATIONENTAL_ij_* 
    + *γ_60_***HELP_SEEKING_ij_* + *γ_61_***SCHTYPE_j_***HELP_SEEKING_ij_* + *γ_62_***URBAN_j_***HELP_SEEKING_ij_* + *γ_63_***SUBURBAN_j_***HELP_SEEKING_ij_* 
     + *u_0j_* + *u_5j_***ENVIRONMENTAL_REGULATION_ij_*  + *u_6j_***HELP_SEEKING_ij_* + *r_ij_*

**BS Use on Math Achievement (Final Model in Table 4):**

*MATH_ACHIEVEMENT_ij_* = *γ_00_* + *γ_01_***SCHTYPE_j_* + *γ_02_***TASK_REGULATION_j_* + *γ_03_***EFFORT_REGULATION_j_* 
    + *γ_04_***TIME_REGULATION_j_* + *γ_05_***ENVIRONMENTAL_REGULATION_j_* + *γ_06_***HELP_SEEKING_j_* + *γ_07_***URBAN_j_* 
    + *γ_08_***SUBURBAN_j_* 
    + *γ_10_***GENDER_ij_* 
    + *γ_20_***TASK_REGULATION_ij_* 
    + *γ_30_***EFFORT_REGULATION_ij_* 
    + *γ_40_***TIME_REGULATION_ij_* 
    + *γ_50_***ENVIRONMENTAL_REGULATION_ij_* 
    + *γ_60_***HELP_SEEKING_ij_* 
    + *u_0j_* + *u_3j_***EFFORT_REGULATION_ij_* + *r_ij_*
