# Supplementary material for: Self-regulated learning strategies and academic achievement in South Korean 6th-graders: A two-level hierarchical linear modeling analysis
Source: PLoS One. 2023 Apr 26;18(4):e0284385. doi: 10.1371/journal.pone.0284385 (PMC10132645; doi:10.1371/journal.pone.0284385)
Supplement: Attachment — Submitted filename: Manuscript_Learning strategies 12032022.docx [file pone.0284385.s002.docx]

**Self-regulated Learning Skills and Academic Achievement in South Korean 6th-graders Based on Varying School Environments**

Cheyeon Ha*^1^, Alysia D. Roehrig^2^, & Qian Zhang ^2^

Yale Child Study Center, School of Medicine, Yale University^1^

Department of Educational Psychology and Learning Systems, Florida State University^2^

**Author Note**

Cheyeon Ha*([cheyeon.ha@yale.edu](mailto:cheyeon.ha@yale.edu)) is a corresponding author of the manuscript and a postdoctoral associate at Yale Child Study Center at Yale University. She contributed to developing research questions and designing research methods and also developed the manuscript.

Dr. Alysia D. Roehrig ([aroehrig@fsu.edu](mailto:aroehrig@fsu.edu)) is a professor in the Learning and Cognition Program at Florida State University. She contributed to providing theoretical foundations for the learning development of school-aged children and to developing the manuscript.

Dr. Qian Zhang ([qzhang4@fsu.edu](mailto:qzhang4@fsu.edu)) is an associate professor in the Measurement and Statistics Program at Florida State University. She contributed to analyzing the secondary datasets and reviewing the data analysis results using hierarchical linear modeling (HLM).

Abstract

The aim of the study was to explain the relationships between self-regulated learning strategy use and academic achievement of 6^th^-grade students in South Korea. An existing database (i.e., the Korean Educational Longitudinal Study; KELS) with 6^th^-grade students (*n* = 7,065) from 446 schools was used to run a series of 2-level hierarchical linear models (HLM). This large dataset enabled us to consider how the relationship between learners’ self-regulated learning strategy use and academic achievement may differ based on environmental factors of school type and school location. We found that students’ metacognition and effort regulation positively predicted their literacy and math achievement both within and across schools. The average literacy and math achievement were significantly higher in private schools than in public schools. Also, the math achievement of urban schools was significantly higher than in non-urban schools when controlling other cognitive and behavioral learning strategies.

***Keywords:*** self-regulated learning (SRL); cognitive strategies; behavioral strategies; early adolescence, school environments

**Self-regulated Learning Skills and Academic Achievement in South Korean 6th-graders Based on Varying School Environments**

Students who can effectively regulate their emotions, thoughts, and behaviors in their learning environments tend to have more advantages in their academic achievement (Linares et al., 2005; Panayiotou et al., 2019; Zeidner et al., 2000). For those reasons, self-regulation skills have been considered an important key to explaining students’ learning success (Stehle & Peters-Burton, 2019). Despite the importance of self-regulation skills in learning, the K-12 curriculum does not often encourage teaching or practicing these skills (Zumbrunn et al., 2011). Most previous studies of learners’ self-regulation of academic achievement have been carried out in the context of higher education; thus, there is still a lack of research on self-regulated learning skills in elementary students. Some evidence-based studies investigated children’s self-regulation abilities at elementary and middle school levels (e.g., Ablard & Lipschultz, 1998; Dent & Koenka, 2016; Fuchs et al., 2003; Guthrie et al., 1999); however, small sample sizes present potential limitations to the generalizability of the prior findings.

Self-regulation has been conceptualized and assessed in the research with various conceptual frameworks, such as emotional regulation, self-control, and self-management. In this study, we focused on the self-regulated learning (SRL) strategies, which are interpreted in a narrow range of cognitive and behavioral strategies commonly observed in students with high self-regulation. For this study, we used national data from the Korean Longitudinal Educational Study (KELS), which offers a large nationally representative sample. This database focuses on young learners’ learning outcomes and relevant psychological factors in K-12 education.

There are various views on the early development of children’s SRL strategies. Dignath et al. (2008) pointed out that SRL strategies can be taught as early as elementary school when students are at an emotional and cognitively fluid state of development. Empirical studies on the development of SRL strategies of children aged 10 through 13 indicate that students' self-regulation skills can be a meaningful factor in explaining their academic performance (Ben-Ari & Eliassy, 2003; Duckworth et al., 2011; Kitsantas et al., 2009). For example, compared to 3^rd^-grade students, 6^th^-grade students are better able to use advanced SRL strategies that enhance their learning (Newman & Schwager, 1995).

In the initial research on SRL, scholars found that self-regulated learners tend to use effective cognitive strategies (e.g., rehearsal, elaboration, organization, and metacognition) and behavioral strategies (e.g., regulation of task, effort, time, environment, and help-seeking) in their learning (Bandura & Wood, 1989; Hofer, 2004; Kitchner, 1983). Pintrich et al.’s (1993) model of self-regulated learning, widely used in SRL studies, comprises both learning strategies and motivational beliefs (i.e., assessed via the Motivated Strategies for Learning Questionnaire; MSLQ). For this study, we used the KELS database, which includes only the cognitive and behavioral learning strategies from Pintrich’s SRL model.

To date, a few studies using the KELS database have been published. For example, researchers investigated the relationship between parents’ and teachers’ expectations and grade 7-9 students’ learning growth (Shin et al., 2016). Another research team found that grade 7-9 students’ intrinsic motivation and self-efficacy moderated the relationship between teachers’ belief and students’ learning growth rates (You et al., 2016). A study by Lim and Lee (2019) compared the patterns of change in students’ career maturity, self-esteem, self-management, and academic achievement from elementary to high school. They found students’ career maturity was a meaningful predictor to self-management skills in elementary school, while high school students’ academic achievement significantly predicted their self-management skills. Most longitudinal studies using the KELS database have focused on how students’ psychological factors and learning outcomes change over time. SRL strategies, however, were assessed at one time point (6th-grade) in the KELS database, and no previous research studies based on KELS data have investigated how learners’ SRL strategies relate to their academic achievement. In this study, we investigated whether self-regulated learning strategy use of 6^th^-grade students predicts their academic achievement; and how school environmental factors moderate the linear relationship between SRL strategy use and academic achievement.

# Background

## Learning Strategies of Self-regulated Learners

Many scholars have theorized that individual differences in self-regulation are key to explaining the learning outcomes of successful learners (Bandura, 1986; Zimmerman, 2008; Zimmerman et al., 1992). SRL scholars explain that self-regulated learners show higher academic performance because they can analyze tasks strategically, set appropriate goals, and manage their thoughts and strategies effectively (Schunk, 2005; Zimmerman, 2000). For example, Murphy and Alexander (2000) theorized that SRL competence is a combination of academic learning and self-control, which, together, raise academic achievement scores because students learn effectively. In contemporary education, using SRL skills to explore information and apply knowledge is becoming increasingly important (Ananiadou & Claro, 2009). This is because an ability to find appropriate information and create meaningful knowledge needs higher level thinking skills. Self-regulated learners tend to use a wider range of strategic skills in learning; thus, previous studies have focused on successful students’ learning strategy use and their academic achievement. Pintrich’s (2000) model describes nine types of SRL strategies, which are categorized as either cognitive strategies or behavioral strategies.

## *Cognitive Strategies (CS) in Learning*

Cognitive psychologists have been focused on how students effectively process more knowledge in working memory and store it in long-term memory. Scholars noted that learners use specific cognitive strategies to build up long-term memory. Moreover, metacognition involves coordinating other cognitive strategies, as a high-level cognitive skill, and it helps students effectively use cognitive strategies in the process of evaluating their learning (Barzilai & Zohar, 2014). Bransford (2000) pointed out that awareness of strengths and limitations in cognitive strategies helps the student learn and cope with learning resources flexibly.

In this study, following Pintrich et al.’s (1993) SRL model, learners’ cognitive learning strategies were organized according to four specific types: 1) rehearsal, 2) elaboration, 3) organization, and 4) metacognition. First, rehearsal strategies are basic-level cognitive skills that load information between working memory and long-term memory. Repetitive exposure involves cognitive processes that help learners recognize important information in working memory and form links in long-term memory (Weinstein et al., 2011). Second, elaboration strategies bridge new information and old knowledge, through processes like paraphrasing and summarizing. Elaboration strategies are processes of transforming information to make it more meaningful and memorable (e.g., application and analysis); thus, elaboration requires more complex cognitive processes compared to rehearsal (Weinstein et al., 2011). Third, organization strategies make meaningful chunks of complex information. Self-regulated learners sense how to reduce complexity and make information well-organized and meaningful. Lastly, metacognitive strategies are higher-level cognitive skills, often defined as thinking about thinking or knowing about knowing (Hofer, 2004; Kitchner, 1983). Students use metacognition for planning, monitoring, and evaluating learning processes.

## *Behavioral Strategies (BS) in Learning*

Since the available resources involved in learning are limited (i.e., time, effort, and environments), learners need to be strategic in their use of behavioral strategies for effective learning. Successful self-regulated learners have been described as tending to use educational resources adequately and to use higher-level of behavioral regulation skills while they are learning (Zimmerman, 2000). According to Pintrich et al.’s (1993) SRL model, learners with high levels of self-regulation show more strategic skills in time-, effort-, and environmental-regulation. Additionally, the model includes help-seeking behaviors, which can enhance collaboration and cooperation and help students better problem-solving in the classroom. Similarly, other scholars theorized that help-seeking skills contribute to increasing productive communications and meaningful learning opportunities that allow students to access scaffolding (Fox & Riconscente, 2008; Williamson, 2015).

In this study, five types of behavioral learning strategies were assessed: task-regulation, effort-regulation, time-regulation, environment-regulation, and help-seeking. First, self-regulated learners use task regulation skills to check their learning progress by preparing, planning, and evaluating task processes (Pintrich, 2000). Second, effort-regulation is a learning skill that helps to maintain endurance even in challenging learning experiences. Learners with high effort-regulation skills can judge and reflect on where effort is most needed. Third, because time is a finite resource, time-regulation ability nurtures learning effectiveness within the planned time (Hensley et al., 2018). Fourth, self-regulated learners have flexibility in external environmental factors; thus they are less affected by external conditions and can even improve learning environments. Finally, a help-seeking approach helps students overcome academic challenges, giving them access to scaffolding from peers and teachers that can enhance their learning (Newman, 2002).

## Self-regulated Learning Strategies and Academic Achievement

Previous empirical studies report that students’ SRL strategies are related to higher academic achievement in college and high school students. For example, Glogger et al. (2012) investigated high school students’ cognitive strategies and found that cognitive learning strategies (i.e., rehearsal, organization, elaboration, and metacognitive skills) were meaningful predictors to explain their journal writing quality. Thiede et al. (2003) reported that supporting metacognition contributed to college students’ higher reading comprehension levels compared to the control group. Multiple studies found that use of self-regulated learning strategies moderates or mediates the relationship between positive academic emotions and academic achievement. For example, a study by Mega et al. (2014) reported that undergraduate students’ SRL use had a positive relationship with their academic achievement, and SRL use also was a meaningful mediator between students’ positive emotions and higher academic achievement. Villavicencio and Bernardo (2013) found that first-year university students’ self-regulation was not a meaningful predictor of their final grade; however, the interaction effect between SRL strategy useself-regulation and enjoyment (e.g., positive emotion) was a significant predictor of their final grade. For example, in a group of students with higher enjoyment, when their SRL strategy use increased, they tended to have higher final grades.

Empirical studies of early childhood and elementary students account for a small percentage of all SRL studies. Some prior studies investigated elementary children’s self-regulated learning strategies and academic performance. A study by Kitsantas et al., (2009) examined 5^th^-grade students’ SRL strategy use, they found that the combined scores of cognitive and behavioral strategies were a significant predictor of students’ achievement in science, language art, and math. These findings are consistent across grade levels and subject areas. For example, Newman and Schwager (1995) found that 6^th^-grade students who frequently used help-seeking strategies in mathematics tended to show higher math achievement. Kindergarten children who effectively regulated self-behavior and exhibited positive classroom behaviors tended to show higher literacy levels (Suchodoletz et al., 2009). Evidence also suggests that the relationship between self-regulation and achievement persists over time. For example, children's behavioral regulation skills at age 7 was a significant predictor of their academic achievement at age 11 (Edossa et al., 2018).

Previous meta-analysis study results of children’s self-regulated learning interventions provide insight into the effect of self-regulation skills on students’ academic performance (Dignath et al., 2008); however, little still is known about elementary students’ SRL strategy use and academic success. Dignath et al. synthesized forty-eight experimental studies of SRL skill training and reported that elementary students showed significant academic improvement in reading/writing and math. In their study, students’ age did not moderate the SRL training effect on academic achievement. All articles analyzed for Diginath et al.’s meta-analysis were conducted in Western cultures. Also, since learning strategy variables were tested as the outcome variables of self-regulation training programs, the results do not provide an understanding of the relationship between academic achievement and the use of specific types of learning strategies.

Another meta-analysis study by Dent and Koenka (2016) synthesized correlational results between cognitive strategy use and students’ academic achievement in elementary and secondary schools. In this meta-analysis, a majority of the correlation studies were performed at the middle and high school levels (*k*= 53, 84%), and studies with elementary students accounted for a small proportion (*k*= 10, 16%). They reported that the overall relationship between cognitive strategy use and higher academic achievement (i.e., science, social studies, and math) was meaningful. However, the correlation study results from elementary students were still unclear because the final results varied depending on different meta-analysis models. For example, the relationship between elementary students’ cognitive strategy use and academic achievement was not meaningful in the random effect model; however, it was negatively correlated with each other in a fixed-effect model. These differences may be due to the imbalanced sample size among the aggregated studies. This means more studies including more samples are needed.

# Study Purpose and Research Questions

This study investigated the relationship between South Korean 6th-graders’ use of SRL strategy use and their academic achievement in literacy and math. Because we assumed that students of this age group are in the early developmental stage of SRL strategies, we hypothesized that students with higher SRL strategy use would show higher academic achievement. In this study, we defined the features of successful self-regulated learning as more frequent use of two types of learning strategies: 1) cognitive strategies and 2) behavioral strategies, as suggested by Pintrich (1993, 2000). The present study answered the following research questions:

Q1. Does 6^th^-grade students’ cognitive learning strategy use predict their academic achievement in literacy and math?

Q2. Does 6^th^-grade students’ behavioral learning strategy use predict their academic achievement in literacy and math?

Q3. Does school type or school location moderate the relationship between 6^th^-grade students’ cognitive strategy use and academic achievement in literacy and math?

Q4. Does school type or school location moderate the relationship between 6^th^-grade students’ behavioral strategy use and academic achievement in literacy and math?

# Method

## Data Sources and Study Design

As secondary data analysis of a national dataset, this study was designed as a cross-sectional study. The Korean Educational Development Institute (KEDI) conducted the national educational data collection project from 2013 through 2018 with a cohort group to track the students’ learning growth. In this study, we used the KELS dataset collected in 2014 with a cohort group of 6^th^-graders, which includes data on students’ SRL strategies. We ran a series of 2-level HLM analyses to test predictors and moderators of students’ academic achievement at the student and school levels.

## Participants

The original KELS dataset was obtained from a total of 7,324 6th-grade students from 458 elementary schools in South Korea. We focused on the 6^th^-graders’ SRL strategy use and academic achievement data from this dataset. After excluding missing data, our final dataset included 7,065 students from 446 schools for the 2-level HLM analyses (Table 1).

[Table 1]

The KELS included similar proportions of gender groups, with 3579 females (50.7%) and 3486 males (49.3%). In the study, gender was set as a dummy variable to compare females' literacy and math achievement (i.e., reference group) against males. The dataset included a total 446 school data from urban (*k* = 181, 40.6%), suburban (*k* = 161, 36.1%), and rural (*k* = 104, 23.3%) areas. Among the schools, 434 schools (*n* = 6717) were public, and 12 schools (*n* = 348) were private schools. These school types and location proportions reflect the general composition of elementary schools in South Korea. Although the number of different school environments was unequal, when groups to be compared have equal or similar variances in the outcome variable, unequal group sizes are not a concern when there is a large enough sample size in both groups (Keppel, 1993).

## Measures

### Students’ cognitive and behavioral learning strategy use was measured using items selected from the MSLQ developed by Pintrich (1993, 2000). Students responded on a 4-point Likert scale (1 = strongly disagree, 4= strongly agree), self-report surveys. Each subscale showed stable reliability levels.

### *Cognitive Learning Strategies*

### The KELS dataset included four cognitive learning strategy subscales with a total of 12-items. The survey assessed *rehearsal* (e.g., “I try to memorize as many things as I can when I study”; 3 items, Cronbach *α* = .78), *elaboration* (e.g., “I try to relate ideas in this subject to those in other subjects whenever possible”; 3 items, Cronbach *α* = .81), *organization* (e.g., “When I study, I try to outline the material to help me organize my thoughts”; 3 items, Cronbach *α* = .82), and *metacognition* (e.g., “When studying for this course I try to determine which concepts I don't understand well.”; 3 items, Cronbach *α* = .86)*.*

### *Behavioral Learning Strategies*

### The KELS dataset included five subscales (17 items total) of behavioral learning strategies: *task-regulation* (e.g., “I find or create my own learning subject or questions”; 5 items, Cronbach *α* = .89), *effort-regulation* (e.g., “Even when subject materials are difficult, I manage to keep working until I finish”; 3 items, Cronbach *α* = .88), *time-regulation* (e.g., “I make good use of my study time for subject learning”; 3 items, Cronbach *α* = .83), *environmental-regulation* (e.g., “I usually study in a place where I can concentrate on my work”; 2 items, Cronbach *α* = .68), and *help-seeking* (e.g., “I ask the teacher to clarify concepts I don't understand well”; 7 items, Cronbach *α*= .84).

### *Academic Achievement in Literacy and Math*

### The KELS database evaluated 6th-grade students' *literacy* and *math achievements* through 25 questions each. The original achievement scores between 0 and 25 were transformed into adjusted scores based on the vertical scaling approach, because it has benefits on the adaptive tests, which measured different questionnaires but focusing on learning growths. The KELS data provide information about students’ academic achievement growth using weights in common questions; thus, the adjusted scores could consider the individual growths as well as test effects. The range of the adjusted *literacy achievement* scores was between 94 and 281 (*M* = 201.49, *SD* = 34.88), and the range of the adjusted *math* scores was between 100 and 267 (*M* = 203.43, *SD* = 39.00).

## Data Analysis

We ran a series of 2-level Hierarchical Linear Modeling (HLM) models to address our research questions, considering both the student-level (level-1) and school-level (level-2) predictors. We investigated four different HLM models based on each of the learning strategies of successful self-regulated learners (i.e., cognitive or behavioral learning strategies) and academic achievement outcomes (i.e., literacy or math). In level-1, the following sub-factors were included in the final models: gender, and either cognitive strategy use (rehearsal, elaboration, organization, and metacognition) or behavioral strategy use (task-, effort-, time-, environment-regulation, and help-seeking). Gender was included as a covariate because it may be related to the linguistic development of school-age children. In general, girls tend to show higher linguistic development in the initial levels compared to boys, and those gender differences in cognitive development may influence their academic achievement of school-age children (Hochweber & Vieluf, 2018; Mohd-Asraf & Abdullah, 2016). In level-2, we tested the moderation effect of the school environments, including school type (i.e., public or private) and location (i.e., urban, suburban, and rural), on the relationships between SRL strategy use and academic achievement in our four initial models.

# Results

First, we obtained the proportion of between-school variability of academic achievement in literacy and math. We set the unconditional model with the outcome variable of academic achievement as below.

*Level 1: (LITERACY; MATH)_ij_ = β_0j_ + r_ij_*

*Level 2: β_0j_ = γ_00_ + u_0j_*

Regarding literacy achievement, the Intraclass Correlation Coefficient (ICC) was 0.143; thus, about 14% of the variability in literacy achievement was between schools. In addition, regarding math achievement, the ICC was .195 or about 19% of the variability in math achievement was between schools. According to the ICC results, we concluded that the multilevel data structure should be accounted for using HLM models.

All the level-1 predictors except *gender* were group-mean centered so that the random effects of these predictors on the literacy and math achievement are within-school (Raudenbush & Bryk, 2002). The reference gender group was set as *female*. We kept variables that had significantly different effects across schools, then added the school environmental variables at level-2 to explain the differences of these effects. For school type, *private* school was set as the reference group. For school location, we set *rural* as the reference group and had two dummies for *urban* and *suburban*. In this study, we estimated four 2-level HLM models using two types of self-regulated learning strategies (i.e., cognitive and behavioral) and achievement in two subjects (i.e., literacy and math). We named the HLM models ‘Cognitive Strategy Literacy’, ‘Cognitive Strategy Math’, ‘Behavioral Strategy Literacy’, and ‘Behavioral Strategy Math’. We describe building the initial and final HLM models in the article, and only report the final HLM model results in the tables.

## Descriptive Statistics

Table 2 shows descriptive statistics of students’ SRL features and academic achievement. For cognitive learning strategies, the averages of the four subscales were similar, about 2.9 on a 4-point Likert scale. In behavioral learning strategies, the means of the subscales were between 2.93 and 3.20, and *effort-regulation* ranked higher than others. The distribution of *math* achievement of 6th-graders showed a greater standard deviation compared to *literacy*. This means that students' individual differences were greater in *math* compared to *literacy*.

[Table 2]

## Cognitive Strategy Use and Academic Achievement

***Cognitive Strategy Literacy Model***

The results of the initial HLM analysis with the cognitive learning strategies as the only predictors showed that *metacognition* positively predicted students’ *literacy* achievement both within and between schools. This suggests that for students in an average school, as *metacognition* increased, their literacy achievement tended to increase, holding constant other predictors (*γ =* 5.657, *p* < .001); also, schools with higher average levels of *metacognition* were associated with higher average levels of *literacy* achievement (*γ =* 13.470, *p* < .01). *Elaboration* and *organization* significantly explained within-school difference, yet not between-school difference in literacy achievement, holding constant other predictors. More specifically, on average across schools, as students’ *elaboration* increased, their literacy achievement also tended to increase (*γ =* 5.079, *p* < .001); in contrast, as students’ *organization* increased, their *literacy* achievement tended to decrease (*γ =* -2.767, *p* < .001), holding constant other predictors. *Rehearsal* did not have a significant between-school effect on *literacy* achievement and its average within-school effect was also non-significant.

Based on the final model, the effects of *gender* and *organization* significantly varied across schools. Thus, we added school-level predictors in the final Cognitive Strategy Literacy Model (Table 3). We found that students’ *literacy* achievement was lower on average in public schools than in private schools (*γ* = -14.276, *p* < .001), holding constant other school-level and student-level predictors. Moreover, *literacy* achievement of *female* students was significantly higher than that of *male* students on average (*γ* = -10.965, *p* < .001), holding constant other school-level and student-level predictors. The directions and tests of between- and within-school effects remained the same as those before adding the school-level predictors.

[Table 3]

***Cognitive Strategy Math Model***

With the same model structure, we investigated how cognitive learning strategies predicted students’ *math* achievement with the initial HLM analysis. *Metacognition* positively predicted students’ *math* achievement both within and between schools. This suggests that for students on average across schools, when their *metacognition* increased, *math* achievement tended to increase, holding constant other predictors (*γ* = 6.323, *p* <.001); also, schools with higher average levels of *metacognition* tended to show higher average levels of *math* achievement (*γ* = 20.234, *p* <.01). *Rehearsal* and *elaboration* significantly explained within-school difference, however, not between-school difference in *math* achievement, holding constant other predictors. On average across schools, when students’ *elaboration* increased, their *math* achievement also tended to increase (*γ* = 6.604, *p* <.001); when students’ *rehearsal* increased, however, their *math* achievement tended to decrease (*γ* = -2.014, *p* < .01), controlling other predictors. *Organization* did not show significant between-school effect or average within-school effect on math achievement. Also, *math* achievement of *male* students was significantly lower than *female* students on average (*γ* = -4.083, *p* < .001), holding constant other school-level and student-level predictors.

The effects of *metacognition* significantly varied across schools. Thus, we added school-level predictors in the final Cognitive Strategy Math Model (Table 3). The results showed that students’ *math* achievement was lower on average in *public* schools than in *private* schools (*γ* = -23.325, *p* < .001), and *urban* schools had higher levels of *math* achievement than *rural* schools (*γ* = 7.00, *p* < .01), holding constant other school-level and student-level predictors. Interestingly, *metacognition* no longer had a significant effect on *math* achievement controlling for school type and school location. The directions and tests of between- and within-school effects of other cognitive learning strategy predictors remained the same as those before adding the school-level predictors.

## Behavioral Strategy Use and Academic Achievement

***Behavioral Strategy Literacy Model***

Among different behavioral learning strategies, only *effort-regulation* positively predicted students’ *literacy* achievement both within and between schools in the initial HLM model. This suggests that on average across schools, as *effort-regulation* increased, students’ *literacy* achievement tended to increase, holding constant other predictors (*γ =* 10.934, *p* < .001); also, schools with higher average levels of *effort-regulation* were related to higher average levels of literacy achievement (*γ =* 16.076, *p* < .001). *Time-regulation* and *environment-regulation* significantly explained within-school difference yet not between-school difference in *literacy* achievement, holding constant other predictors. More specifically, on average across schools, when students’ *time-regulation* increased, their *literacy* achievement tended to decrease (*γ =* -7.85, *p* < .001); in contrast, as students’ *environment-regulation* increased, *literacy* achievement tended to increase (*γ =* 4.514, *p* < .001), holding constant other predictors. *Help-seeking* and *task-regulation* had significant between-school effects. Schools with lower levels of *help-seeking* and higher levels of *task-regulation* tended to have higher levels of *literacy* achievement, holding constant other predictors. Moreover, *literacy* achievement of *males* was significantly lower than that of *females* on average (*γ* = -7.308, *p* < .001), holding constant other school-level and student-level predictors.

The effects of *environment-regulation* and *help-seeking* significantly varied across schools. Thus, we added school-level predictors in the final Behavioral Strategy Literacy Model (Table 4). We found that students’ *literacy* achievement was lower on average in *public* schools than in *private* schools (*γ* = -12.520, *p* < .001), holding constant other school-level and student-level predictors. Furthermore, within-school effects of *help-seeking* varied by school locations. Specifically, students in *urban* and *suburban* schools had positive average effects whereas those in *rural* schools had a negative average effect on *literacy* achievement, controlling for school type and other student-level predictors.

[Table 4]

***Behavioral Strategy Math Model***

In the initial HLM model of how students’ behavioral learning strategies predicted their *math* achievement, e*ffort-regulation* predicted students’ *math* achievement both within and between schools. This means that on average across schools, when students had higher *effort-regulation*, their *math* achievement tended to increase, holding constant other predictors (*γ* = 12.194, *p* < .001); also, schools with higher average levels of *effort-regulation* tended to show higher average levels of *math* achievement (*γ* = 18.384, *p* < .01). *Time-regulation* and *environment-regulation* also significantly explained within-school difference yet not between-school difference in *math* achievement, holding constant other predictors. On average across schools, when students’ *environment-regulation* increased, their *math* achievement also tended to increase (*γ* = 5.382, *p* < .001), but when students’ *time-regulation* increased, their *math* achievement tended to decrease (*γ* = -2.266, *p* < .05), while controlling for other predictors. *Help-seeking* did not have a significant within-school effect, but it had a significant between-school effect (*γ* = -12.330, *p* < .05) on *math* achievement. Also, *math* achievement of *male* students was significantly lower than *female* students on average (*γ* = -2.953, *p* < .01), holding constant other school-level and student-level predictors.

The within-school effect of *effort-regulation* significantly varied across schools. Thus, we added school-level predictors in the final Behavioral Strategy Math Model (Table 4). The results showed that students’ *math* achievement was lower on average in *public* schools than in *private* schools (*γ* = -23.509, *p* < .001), holding constant other school-level and student-level predictors. In addition, *urban* schools had higher *math* achievement than *rural* schools controlling for other predictors (*γ* = 5.409, *p* < .05). The directions and tests of between- and within-school effects remained the same as those before adding the school-level predictors.

# Discussion

Twenty-first century learners need to have learning opportunities to activate various knowledge such as procedural knowledge (e.g., how to use the learning strategies) and conditional knowledge (when and why to use learning various strategies in a context) (Schunk, 2005). The results of investigating the SRL strategy use of 6th-grade South Korean students highlights the differences between successful adult learners and developing young learners, as developmental issues.

In many previous studies, scholars have highlighted the important role of metacognition in controlling effective cognitive processing through planning, monitoring, and evaluation in the late adolescent and adult years (Barzilai & Zohar, 2014; Hofer, 2004; Kim et al., 2015; Kitchner, 1983). This study’s findings additionally support that South Korean elementary students’ metacognitive strategy use is an important learning skill supporting academic performance in literacy and math. Students who review their thinking processes in subject learning may solve problems more effectively under the same learning environment. Moreover, students in the same school environment may tend to show higher academic performance as they more frequently use elaborative cognitive strategies.

In this study, we found that students’ rehearsal strategy use negatively predicted math achievement, controlling for other cognitive strategy use in an average school. A meta-analysis study by Broadbent and Poon (2015) reported that metacognition predicts university students’ higher achievement; however, elaboration and rehearsal were not. Moreover, Weinstein et al. (2011) pointed out that rehearsal, as a lower level cognitive strategy, may show a different pattern in relation to achievement compared to other higher level cognitive strategies. For example, in simple problem-solving tasks in math, frequent use of rehearsal may increase cognitive load, thus reducing learning efficiency. As in our sample, students with higher math skills may use fewer rehearsal strategies.

Regarding students’ use of behavioral learning strategies, we found that students with higher academic achievement tended to regulate their effort depending on task difficulty. Kim et al. (2015) reported similar results at the high school level, such that students’ effort-regulation was related to their academic achievement, with this linear relationship stronger in a group of high achievers. However, in this study, we found use of time-regulation and help-seeking strategies did not predict better achievement. A meta-analysis study by Broadbent and Poon (2015) showed that metacognition, time-regulation, and effort-regulation related to the higher achievement of university students in various online learning environments.

The relationship between students’ help-seeking and achievement needs to be interpreted considering the various learning contexts. For example, Broadbent and Poon’s (2015) meta-analysis results indicate that the effect of help-seeking on students’ achievement varies; the aggregated results, from twelve different empirical studies, showed that help-seeking did not consistently predict students’ achievement; thus, it may depend on various learning settings. In this study, South Korean students’ help-seeking was negatively related to math achievement. Related to the cultural background, Loh and Teo (2017) pointed out that in Eastern culture, students tend to be more passive learners and feel difficulties asking questions to others when they need help.

We found that South Korean urban school students had higher math achievement when controlling all other learning strategies and gender differences; however, there were no differences in literacy related to school location. Also, students in private schools showed higher achievement in literacy and math, when controlling all other learning strategies. In the cognitive strategy models, the relation between the four cognitive strategies and achievement did not vary depending on school environmental factors. However, in the behavioral strategy model, the relationship between help-seeking strategy and literacy achievement varied in relation to school location. We found a stronger effect of help-seeking for students at urban schools, such that using help-seeking strategies predicts better literacy achievement.

In this study, females had better academic performance both in literacy and math achievement. In prior studies, scholars have indicated that gender differences in academic achievement may be caused by different growth patterns in children’s linguistic and cognitive development (Hochweber & Vieluf, 2018; Mohd-Asraf & Abdullah, 2016). On the other hand, it is well known that females at the high school and undergraduate levels show lower preference for STEM (i.e., Science, Technology, Engineering, and Mathematics) majors and higher anxiety about learning in it (Legewie & DiPrete, 2014; Liben & Coyle, 2014). Our results support the previous findings that students’ STEM performance is not simply explained by gender differences; there are complex relevant factors, such as differences in teachers’/parents’ expectation (Crowley et al., 2001; Newcombe & Frick, 2010) and extracurricular experiences (Jones et al., 2000), that come into play.

**Limitations and Future Research**

As a cross-sectional study, our findings explain how students’ current SRL strategies were related to their current academic achievement. However, the results of the study partly demonstrated that the use of self-regulated learning strategies by 6^th^-grade students is not entirely identical to the pattern seen in adult learners. In this study, for elementary students, some learning strategies are not predictive of achievement. However, considering previous studies results with university-level students (Puzziferro, 2008; Wang & Wu, 2008), young students need to learn specific learning strategies (e.g., time-regulation, elaboration, and organization) that were not predictive in this study to prepare for more advanced learning in the future. This suggests that elementary learners may need to experience more opportunities for learning self-regulating learning strategies in school curricula. Longitudinal follow-up studies are needed to answer questions about the development of elementary students’ use of SRL strategies into middle and high school.

When we conducted preliminary analyses, we found the four cognitive strategies and five behavioral strategies were highly correlated with each other. To avoid multicollinearity issues and also to build a parsimonious model avoiding high-complexity in the final model, we decided to build two separate HLM models for cognitive strategies and for behavioral strategies. Pintrich and colleagues (1993), the originators of the SRL model, also estimated cognitive and behavioral strategies separately. These were considerations in our final HLM analysis decision. Future research could focus on the relationships between cognitive and behavioral strategies to determine whether and how those factors influence each other. Path analysis results among cognitive and behavioral strategies could provide a greater understanding of how those different strategies can be interconnected and developed together.

In this study, we estimated school environmental factors such as school types and locations. This study’s findings showed that there was a notable achievement gap between public and private schools in South Korea. The average scores of literacy and math were both high in private schools. This may be related to the socioeconomic status issue rather than the different curriculums in specific school types. In South Korea, a high proportion of low achieving students belong to low-income families (OECD, 2018). The majority of elementary schools belong to the public sector, where the government provides free educational services. Private elementary school students have a relatively better socioeconomic background than public elementary school students (Byun, 2017). From those reports, it is notable that students in poverty mostly enter public elementary schools. Further studies need to consider students’ socioeconomic backgrounds to explain the gaps in achievement and learning strategies. Future research in this area would serve an important social justice purpose if it can help address inequity.

**Conclusion**

SRL strategy use is one of the most important learning skills for future generations. Our results raise important implications for future directions for self-regulation research and practice in K-12 education. This study suggests that elementary students should have learning opportunities to expand their use of SRL strategies and grow into successful adult learners. Schools should provide an appropriate learning experience to help students develop these skills well. For example, relevant educational studies, including this study, agree on the importance of supporting metacognitive strategies in learning. Thus, teachers need to help children learn to plan, monitor, and evaluate their learning processes during classroom activities. Educational researchers need to cooperate closely with teachers to incorporate these theoretical principles into specific subject curricula. Teachers' roles, guiding SRL strategies, should be better supported in school environments, including public schools and rural areas, where achievement gaps are evident. School location was an influential factor in mathematical achievement, although there was no direct effect on literacy achievement. This suggests there is a particular need to support SRL strategies for rural students in math education, as it might be a potential problem for rural students to prepare for future STEM education. Promoting metacognition, taks-regulation, and effort-regulation learning strategies could help young students to increase their learning efficiency.

**Acknowledgements**

We have no known conflict of interest to disclose, and no funding was received for this work. We used secondary data resources from the Korean national database and were authorized to use data by the Korean Educational Development Institute (KEDI). As a secondary data analysis study, the IRB process of this study was officially exempted by confirmation from the FSU IRB Committee.

**References**

Ablard, K. E., & Lipschultz, R. E. (1998). Self-regulated learning in high-achieving students: Relations to advanced reasoning, achievement goals, and gender. *Journal of Educational Psychology, 90*(1), 94-101. <https://doi.org/10.1037/0022-0663.90.1.94>

Ananiadou, K., & Claro, M. (2009). 21st century skills and competences for new millennium Learners in OECD Countries. <https://doi.org/10.1787/218525261154>

Bandura, A. (1986). *Social foundations of thought and action: A social cognitive theory*. Prentice Hall.

Bandura, A., & Wood, R. (1989). Effect of perceived controllability and performance standards on self-regulation of complex decision making. *Journal of Personality and Social Psychology, 56*(5), 805-814.

Barzilai, S., & Zohar, A. (2014). Reconsidering personal epistemology as metacognition: A multifaceted approach to the analysis of epistemic thinking. *Educational Psychologist*, *49*(1), 13-35. <https://doi.org/10.1080/00461520.2013.863265>

Ben-Ari, R., & Eliassy, L. (2003). The differential effects of the learning environment on student achievement motivation: A comparison between frontal and complex instruction strategies. *Social Behavior and Personality, 31*(2), 143-165. <http://doi/org/10.2224/sbp.2003.31.2.143>

Bransford, J. D., Brown Ann L., and Cocking Rodney R. (2000). *How people learn: Brain, mind, experience, and school*. National Academy Press.

Broadbent, J., & Poon, W. L. (2015). Self-regulated learning strategies and academic achievement in online higher education learning environments: A systematic review. *The Internet and Higher Education*, *27*, 1-13. <https://doi.org/10.1016/j.iheduc.2015.04.007>

Byun, S. Y. (2017). Differences in academic achievement between private and public elementary school graduates during the middle school years. *Korean Journal of Educational Research*, *55*(4), 167-187.

Crowley, K., Callanan, M. A., Tenenbaum, H. R., & Allen, E. (2001).Parents explain more often to boys than to girls during shared scientific thinking. *Psychological Science*, *12*, 258–261. <http://dx.doi.org/10.1111/1467-9280.00347>

Dent, A. L., & Koenka, A. C. (2016). The Relation between self-regulated learning and academic achievement across childhood and adolescence: A meta-analysis. *Educational Psychology Review, 28*(3), 425-474. <https://doi.org/10.1007/s10648-015-9320-8>

Dignath, C., Buettner, G., & Langfeldt, H.-P. (2008). How can primary school students learn self-regulated learning strategies most effectively?: A meta-analysis on self-regulation training programmes. *Educational Research Review, 3*(2), 101-129. <https://doi.org/10.1016/j.edurev.2008.02.003>

Duckworth, A. L., Kirby, T. A., Tsukayama, E., Berstein, H., & Ericsson, K. A. (2011). Deliberate practice spells success: Why grittier competitors triumph at the National Spelling Bee. *Social Psychological and Personality Science, 2*(2), 174-181. <https://doi.org/10.1177/1948550610385872>

Eccles, J. S., & Wigfield, A. (2002). Motivational beliefs, values, and goals. *Annual Review of Psychology, 53*(1), 109-132. <http://doi.org/10.1146/annurev.psych.53.100901.135153>

Edossa, A. K., Schroeders, U., Weinert, S., & Artelt, C. (2018). The development of emotional and behavioral self-regulation and their effects on academic achievement in childhood. *International Journal of Behavioral Development, 42*(2), 192-202. <https://doi.org/10.1177/0165025416687412>

Fox, E., & Riconscente, M. (2008). Metacognition and self-regulation in James, Piaget, and Vygotsky. *Educational Psychology Review, 20*(4), 373-389. <https://doi.org/10.1007/s10648-008-9079-2>

Fuchs, L. S., Fuchs, D., Prentice, K., Burch, M., Hamlett, C. L., Owen, R., & Schroeter, K. (2003). Enhancing third-grade student' mathematical problem solving with self-regulated learning strategies. *Journal of Educational Psychology, 95*(2), 306-315. <https://doi.org/10.1037/0022-0663.95.2.306>

Glogger, I., Schwonke, R., Holzäpfel, L., Nückles, M., & Renkl, A. (2012). Learning strategies assessed by journal writing: Prediction of learning outcomes by quantity, quality, and combinations of learning strategies. *Journal of Educational Psychology, 104*(2), 452-468. <https://doi.org/10.1037/a0026683>

Guthrie, J. T., Anderson, E., Alao, S., & Rinehart, J. (1999). Influences of concept-oriented reading instruction on strategy use and conceptual learning from text. *The Elementary School Journal, 99*(4), 343-366. <https://doi.org/10.1086/461929>

Hensley, L. C., Wolters, C. A., Won, S., & Brady, A. C. (2018). Academic probation, time management, and time use in a college success course. *Journal of College Reading and Learning, 48*(2), 105-123. <https://doi.org/10.1080/10790195.2017.1411214>

Hochweber, J., & Vieluf, S. (2018). Gender differences in reading achievement and enjoyment of reading: The role of perceived teaching quality. *The Journal of Educational Research, 111*(3), 268-283. <https://doi.org/10.1080/00220671.2016.1253536>

Hofer, B. K. (2004). Epistemological understanding as a metacognitive process: Thinking aloud during online searching. *Educational Psychologist, 39*(1), 43-55. <https://doi.org/0.1207/s15326985ep3901_5>

Jones, M. G., Howe, A., & Rua, M. J. (2000). Gender differences instudents’ experiences, interests, and attitudes toward science and scientists. *Science Education*, *84*, 180–192. <http://dx.doi.org/10.1002/>

Keppel, G. (1993). *Design and analysis: A researcher’s handbook*. Pearson.

Kim, C., Park, S. W., Cozart, J., & Lee, H. (2015). From motivation to engagement: The role of effort regulation of virtual high school students in mathematics courses. *Journal of Educational Technology & Society*, *18*(4), 261-272.

Kitchner, K. S. (1983). Cognition, metacognition, and epistemic cognition. *Human development, 26*(4), 222-232. <https://doi.org/10.1159/000272885>

Kitsantas, A., Steen, S., & Huie, F. (2009). The role of self-regulated strategies and goal orientation in predicting achievement of elementary school children. *International Electronic Journal of Elementary Education, 2*(1), 65-81.

Legewie, J., & DiPrete, T. A. (2014). The high school environment and the gender gap in science and engineering. *Sociology of Education, 87*(4), 259-280. <https://doi.org/10.1177/0038040714547770>

Liben, L. S., & Coyle, E. F. (2014). Chapter three developmental interventions to address the STEM gender gap: Exploring intended and unintended consequences. In L. S. Liben & R. S. Bigler (Eds.), *Advances in Child Development and Behavior* (Vol. 47, pp. 77-115). JAI. <https://doi.org/10.1016/bs.acdb.2014.06.001>

Lim, H.-J., & Lee, S.-B. (2019). Student and school characteristics influencing self-management ability as future key competency among elementary and high school students. *The Journal of Child Education, 28*(1), 73-95. <https://doi.org/10.17643/KJCE.2019.28.1.05>

Linares, L. O., Rosbruch, N., Stern, M. B., Edwards, M. E., Walker, G., Abikoff, H. B., & Alvir, J. M. J. (2005). Developing cognitive-social-emotional competencies to enhance academic learning. *Psychology in the Schools, 42*(4), 405-417. <https://doi.org/10.1002/pits.20066>

Loh, C. Y. R., & Teo, T. C. (2017). Understanding Asian students learning styles, cultural influence and learning strategies. *Journal of Education & Social Policy, 7*(1), 194-210.

Mega, C., Ronconi, L., & De Beni, R. (2014). What makes a good student? How emotions, self-regulated learning, and motivation contribute to academic achievement. *Journal of Educational Psychology, 106*(1), 121-131. <http://dx.doi.org/10.1037/a0033546>

Mohd-Asraf, R., & Abdullah, H. (2016). Elementary schoolers' attitudes toward reading in English: How boys feel relative to girls. *English Language Teaching, 9*(6), 134-140. <http://doi.org/10.5539/elt.v9n6p134>

Murphy, P. K., & Alexander, P. A. (2000). A motivated exploration of motivation terminology. *Contemporary Educational Psychology, 25*(1), 3-53. <https://doi.org/10.1006/ceps.1999.1019>

Newcombe, N. S., & Frick, A. (2010). Early education for spatial intelli-gence: Why, what, and how. *Mind, Brain, and Education*, *4*, 102–111. <http://dx.doi.org/10.1111/j.1751-228X.2010.01089.x>

Newman, R. S. (2002). How self-regulated learners cope with academic difficulty: The role of adaptive help seeking. *Theory Into Practice, 41*(2), 132-138. <https://doi.org/10.1207/s15430421tip4102_10>

Newman, R. S., & Schwager, M. T. (1995). Students’ help seeking during problem solving: Effects of grade, goal, and prior achievement. *American Educational Research Journal, 32*(2), 352-376. <https://doi.org/10.3102/00028312032002352>

OECD (2019), *PISA 2018 Results (Volume I): What Students Know and Can Do*, PISA, OECD Publishing. <https://doi.org/10.1787/5f07c754-en>

Panayiotou, M., Humphrey, N., & Wigelsworth, M. (2019). An empirical basis for linking social and emotional learning to academic performance. *Contemporary Educational Psychology*, *56*, 193-204. <https://doi.org/10.1016/j.cedpsych.2019.01.009>

Pintrich, P. R. (2000). The role of goal orientation in self-regulated learning. In M. Z. Boekaerts Monique, Paul R. Pintrich (Ed.), *Handbook of Self-regulation* (pp. 451-502). Academic Press.

Pintrich, P. R., Smith, D. A., Garcia, T., & McKeachie, W. J. (1993). Reliability and predictive validity of the Motivated Strategies for Learning Questionnaire (MSLQ). *Educational and Psychological Measurement, 53*(3), 801-813. <https://doi.org/10.1177/0013164493053003024>

Puzziferro, M. (2008). Online technologies self-efficacy and self-regulated learning as predictors of final grade and satisfaction in college-level online courses. *American Journal of Distance Education*, *22*(2), 72–89. <https://doi.org/10.1080/08923640802039024>

Schunk, D. H. (2005). Self-regulated learning: The educational legacy of Paul R. Pintrich. *Educational Psychologist, 40*(2), 85-94. <https://doi.org/10.1207/s15326985ep4002_3>

Shin, J., Seo, E., & Hwang, H. (2016). The effects of social supports on changes in students' perceived instrumentality of schoolwork for future goal attainment. *Educational Psychology, 36*(5), 1024-1043. <https://doi.org/10.1080/01443410.2015.1072135>

Stehle, S. M., & Peters-Burton, E. E. (2019). Developing student 21 st Century skills in selected exemplary inclusive STEM high schools. *International Journal of STEM Education, 6*(1), 1-15. <https://doi.org/10.1186/s40594-019-0192-1>

Suchodoletz, A., Trommsdorff, G., Heikamp, T., Wieber, F., & Gollwitzer, P. M. (2009). Transition to school: The role of kindergarten children's behavior regulation. *Learning and Individual Differences, 19*(4), 561-566. <https://doi.org/10.1016/j.lindif.2009.07.006>

Thiede, K. W., Anderson, M., & Therriault, D. (2003). Accuracy of metacognitive monitoring affects learning of texts. *Journal of Educational Psychology, 95*(1), 66-73. <http://dx.doi.org/10.1037/0022-0663.95.1.66>

Villavicencio, F. T., & Bernardo, A. B. (2013). Positive academic emotions moderate the relationship between self‐regulation and academic achievement. *British Journal of Educational Psychology, 83*(2), 329-340. <https://doi.org/10.1111/j.2044-8279.2012.02064.x>

Wang, S. L., & Wu, P.Y. (2008). The role of feedback and self-efficacy on web-based learning: the social cognitive perspective. *Computers & Education*, *51*(4), 1589–1598. <https://doi.org/10.1016/j.compedu.2008.03.004>

Weinstein, C. E., Acee, T. W., & JaeHak, J. (2011). Self-regulation and learning strategies. *New Directions for Teaching & Learning, 126*, 45-53. <https://doi.org/10.1002/tl.443>

Williamson, G. (2015). Self-regulated learning: an overview of metacognition, motivation and behaviour. *Journal of Initial Teacher Inquiry, 1*, 25-27. <http://dx.doi.org/10.26021/851>

You, S., Dang, M., & Lim, S. (2016). Effects of student perceptions of teachers' motivational behavior on reading, English, and mathematics achievement: The mediating role of domain specific self-efficacy and intrinsic motivation. *Child & Youth Care Forum, 45*(2), 221-240. <https://doi.org/10.1007/s10566-015-9326-x>

Zeidner, M., Boekaerts, M., & Pintrich, P. R. (2000). *Self-regulation: Directions and challenges for future research.* In *Handbook of Self-regulation* (pp. 749-768). Academic Press.

Zimmerman, B. J. (2000). Self-efficacy: An essential motive to learn. *Contemporary Educational Psychology, 25*(1), 82-91. <https://doi.org/10.1006/ceps.1999.1016>

Zimmerman, B. J. (2008). Investigating self-regulation and motivation: Historical background, methodological developments, and future prospects. *American Educational Research Journal, 45*(1), 166-183. <https://doi.org/10.3102/0002831207312909>

Zimmerman, B. J., Bandura, A., & Martinez-Pons, M. (1992). Self-motivation for academic attainment: The role of self-efficacy beliefs and personal goal setting. *American Educational Research Journal, 29*(3), 663-676. <https://doi.org/10.3102/00028312029003663>

Zumbrunn, S., Tadlock, J., & Roberts, E. D. (2011). Encourage Self regulated learning in the classroom. *Metropolitan Educational Research Consortium (MERC) Publication*, 1-28. Retrieved from <http://scholarscompass.vcu.edu/merc_pubs/18>

# Tables

**Table 1**

*Demographic Information of Participants*

| Variables | Categories | Sample size | Proportion (%) |
| --- | --- | --- | --- |
| Gender (*n* = 7065) | Female | 3579 | 50.7 |
|  | Male | 3486 | 49.3 |
| School types (*k* = 446) | Public | 434 | 97.3 |
|  | Private | 12 | 2.7 |
| School locations (*k* = 446) | Urban | 181 | 40.6 |
|  | Suburban | 161 | 36.1 |
|  | Rural | 104 | 23.3 |

**Table 2**

*Descriptive Statistics* (*n* = 7,065)

| Variables | Subscales | *Min*. | *Max*. | *M* | *SD*s |
| --- | --- | --- | --- | --- | --- |
| Cognitive | Rehearsal | 1.00 | 4.00 | 2.96 | 0.57 |
| Learning- | Elaboration | 1.00 | 4.00 | 2.91 | 0.63 |
| Strategy Use | Organization | 1.00 | 4.00 | 2.92 | 0.66 |
|  | Metacognition | 1.00 | 4.00 | 2.93 | 0.66 |
| Behavioral | Task-regulation | 1.00 | 4.00 | 2.93 | 0.62 |
| Learning- | Effort-regulation | 1.00 | 4.00 | 3.20 | 0.58 |
| Strategy Use | Time-regulation | 1.00 | 4.00 | 2.82 | 0.64 |
|  | Environmental-regulation | 1.00 | 4.00 | 3.02 | 0.62 |
|  | Help-seeking | 1.00 | 4.00 | 2.94 | 0.54 |
| Achievement | Literacy | 94.00 | 281.00 | 201.49 | 34.89 |
|  | Math | 100.00 | 267.00 | 203.43 | 39.00 |

**Table 3**

*HLM Results of Cognitive Strategy Use and Academic Achievement*

|  | Cognitive Strategy Literacy  Final Model | | | Cognitive Strategy Math  Final Model | | |
| --- | --- | --- | --- | --- | --- | --- |
| Fixed Effect | *B* | *SD*s | *p* | *B* | *SD*s | *p* |
|  |  |  |  |  |  |  |
| For INTRCPT1, *β_0_* | | | |  | | |
| INTRCPT2 | 190.226 | 13.728 | <0.001*** | 190.962 | 16.725 | <0.001*** |
| SCHOOL TYPE | -14.276 | 2.113 | <0.001*** | -23.325 | 2.330 | <0.001*** |
| REHEARSAL | -2.304 | 2.893 | 0.426 | -5.589 | 3.649 | 0.126 |
| ELABORATION | -1.826 | 5.146 | 0.723 | 3.603 | 6.381 | 0.573 |
| ORGANIZATION | 2.291 | 4.536 | 0.614 | -2.471 | 5.733 | 0.667 |
| METACOGNITION | 11.435 | 4.685 | 0.015* | 16.072 | 5.602 | 0.004** |
| URBAN | 1.862 | 2.125 | 0.382 | 6.997 | 2.198 | 0.002** |
| SUBURBAN | 1.576 | 2.073 | 0.448 | 0.116 | 2.259 | 0.959 |
| For GENDER slope, *β_1_* | | | |  | | |
| INTRCPT2 | -10.965 | 2.960 | <0.001*** | -4.083 | 0.953 | <0.001*** |
| SCHOOL TYPE | 3.899 | 2.616 | 0.137 |  |  |  |
| URBAN | 0.738 | 2.239 | 0.742 |  |  |  |
| SUBURBAN | -3.479 | 2.464 | 0.159 |  |  |  |
| For REHEARSAL slope, *β_2_* | | | |  | | |
| INTRCPT2 | -1.126 | 0.587 | 0.055 | -2.035 | 0.676 | 0.003** |
| For ELABORATION slope, *β_3_* | | | |  | | |
| INTRCPT2 | 5.102 | 0.964 | <0.001*** | 6.613 | 1.053 | <0.001*** |
| For ORGANIZATION slope, *β_4_* | | | |  | | |
| INTRCPT2 | -5.500 | 2.778 | 0.048* | -1.602 | 1.052 | 0.128 |
| SCHOOL TYPE | 3.597 | 2.631 | 0.172 |  |  |  |
| URBAN | -1.563 | 1.580 | 0.323 |  |  |  |
| SUBURBAN | -0.093 | 1.736 | 0.957 |  |  |  |
| For METACOGNITION slope, *β_5_* | | | |  | | |
| INTRCPT2 | 5.677 | 0.976 | <0.001*** | 1.000 | 2.778 | 0.719 |
| SCHOOL TYPE |  |  |  | 3.946 | 2.656 | 0.138 |
| URBAN |  |  |  | 2.690 | 1.687 | 0.112 |
| SUBURBAN |  |  |  | 0.955 | 1.865 | 0.609 |

*Note*. Final estimates were calculated based on the robust standard error. * *p* < 0.05, ** *p* < 0.01, and ****p* < 0.001.

**Table 4**

*HLM Results of Behavioral Strategy Use and Academic Achievement*

|  | Behavioral Strategy Literacy  Final Model | | | Behavioral Strategy Math  Final Model | | |
| --- | --- | --- | --- | --- | --- | --- |
| Fixed Effect | *B* | *SD*s | *p* | *B* | *SD*s | *p* |
| For INTRCPT1, *β_0_* | | | |  |  |  |
| INTRCPT2 | 175.262 | 13.574 | <0.001*** | 161.775 | 14.682 | <0.001*** |
| SCHOOL TYPE | -12.521 | 2.346 | <0.001*** | -23.509 | 2.414 | <0.001*** |
| TASK REGULATION | 10.663 | 4.652 | 0.022* | 13.240 | 6.639 | 0.047* |
| EFFORT REGULATION | 15.266 | 4.925 | 0.002** | 16.621 | 6.366 | 0.009** |
| TIME REGULATION | -7.711 | 4.450 | 0.084 | -5.525 | 5.297 | 0.352 |
| ENVIRONMENT REGULATION | 4.176 | 4.338 | 0.336 | 7.721 | 5.378 | 0.152 |
| HELP SEEKING | -9.946 | 5.073 | 0.051 | -12.328 | 5.787 | 0.034* |
| URBAN | 1.344 | 2.016 | 0.505 | 5.409 | 2.180 | 0.013* |
| SUBURBAN | -0.627 | 1.930 | 0.746 | -0.789 | 2.205 | 0.721 |
| For GENDER slope, *β_1_* | | | |  |  |  |
| INTRCPT2 | -7.308 | 0.824 | <0.001*** | -2.953 | 0.902 | 0.001** |
| For TASK REGULATION slope, *β_2_* | | | |  |  |  |
| INTRCPT2 | 0.4383 | 0.9440 | 0.642 | 0.217 | 1.037 | 0.834 |
| For EFFORT REGULATION slope, *β_3_* | | | |  |  |  |
| INTRCPT2 | 10.919 | 1.077 | <0.001*** | 4.123 | 3.387 | 0.283 |
| SCHOOL TYPE |  |  |  | 5.428 | 3.652 | 0.138 |
| URBAN |  |  |  | 4.037 | 2.180 | 0.065 |
| SUBURBAN |  |  |  | 3.209 | 2.415 | 0.185 |
| For TIME REGULATION slope, *β_4_* | | | |  |  |  |
| INTRCPT2 | -7.870 | 0.965 | <0.001*** | -2.288 | 1.092 | 0.036* |
| For ENVIRONMENTAL REGULATION slope, *β_5_* | | | |  |  |  |
| INTRCPT2 | 2.477 | 3.643 | 0.497 | 5.360 | 0.938 | <0.001*** |
| SCHOOL TYPE | 1.851 | 3.191 | 0.562 |  |  |  |
| URBAN | -0.384 | 2.094 | 0.854 |  |  |  |
| SUBURBAN | 1.121 | 2.343 | 0.633 |  |  |  |
| For HELP_SEEKING slope, *β_6_* | | | |  |  |  |
| INTRCPT2 | -3.538 | 5.081 | 0.487 | -1.348 | 1.167 | 0.248 |
| SCHOOL TYPE | 0.726 | 4.691 | 0.877 |  |  |  |
| URBAN | 5.797 | 2.411 | 0.017* |  |  |  |
| SUBURBAN | 5.077 | 2.427 | 0.037* |  |  |  |

*Note*. Final estimates were calculated based on the robust standard error. * *p* < 0.05, ** *p* < 0.01, and ****p* < 0.001.
