# Supplementary material for: Self-regulated learning strategies and academic achievement in South Korean 6th-graders: A two-level hierarchical linear modeling analysis
Source: PLoS One. 2023 Apr 26;18(4):e0284385. doi: 10.1371/journal.pone.0284385 (PMC10132645; doi:10.1371/journal.pone.0284385)
Supplement: Attachment — Submitted filename: Response to Reviewers_03082023_final.docx [file pone.0284385.s003.docx]

ID: PONE-D-22-33255

Title: Self-regulated learning strategies and academic achievement in South Korean 6th-graders: A two-level hierarchical linear modeling analysis
---------------------------------------------

Dear Authors,

You are required to address reservations raised by each reviewer. Be specific in your response and indicate changes in the manuscript.

------------------------------------------------
Reviewers' comments:

Reviewer's Responses to Questions

**Comments to the Author**

1. Is the manuscript technically sound, and do the data support the conclusions?

The manuscript must describe a technically sound piece of scientific research with data that supports the conclusions. Experiments must have been conducted rigorously, with appropriate controls, replication, and sample sizes. The conclusions must be drawn appropriately based on the data presented.

Reviewer #1: Yes

Reviewer #2: No

Reviewer #3: Partly

Reviewer #4: Yes

2. Has the statistical analysis been performed appropriately and rigorously?

Reviewer #1: Yes

Reviewer #2: Yes

Reviewer #3: Yes

Reviewer #4: Yes

3. Have the authors made all data underlying the findings in their manuscript fully available?

The [PLOS Data policy](https://nam12.safelinks.protection.outlook.com/?url=http%3A%2F%2Fwww.plosone.org%2Fstatic%2Fpolicies.action%23sharing&data=05%7C01%7Ccheyeon.ha%40yale.edu%7C73d1f484e721430a3f7208db03ae38af%7Cdd8cbebb21394df8b4114e3e87abeb5c%7C0%7C0%7C638107819491637299%7CUnknown%7CTWFpbGZsb3d8eyJWIjoiMC4wLjAwMDAiLCJQIjoiV2luMzIiLCJBTiI6Ik1haWwiLCJXVCI6Mn0%3D%7C3000%7C%7C%7C&sdata=lRTALJQvSleiqxqx32PI8FzF7WV5uwEdiO1JNVOkmDY%3D&reserved=0) requires authors to make all data underlying the findings described in their manuscript fully available without restriction, with rare exception (please refer to the Data Availability Statement in the manuscript PDF file). The data should be provided as part of the manuscript or its supporting information, or deposited to a public repository. For example, in addition to summary statistics, the data points behind means, medians and variance measures should be available. If there are restrictions on publicly sharing data—e.g. participant privacy or use of data from a third party—those must be specified.

Reviewer #1: Yes

Reviewer #2: Yes

Reviewer #3: Yes

Reviewer #4: Yes

4. Is the manuscript presented in an intelligible fashion and written in standard English?

PLOS ONE does not copyedit accepted manuscripts, so the language in submitted articles must be clear, correct, and unambiguous. Any typographical or grammatical errors should be corrected at revision, so please note any specific errors here.

Reviewer #1: Yes

Reviewer #2: No

Reviewer #3: Yes

Reviewer #4: Yes

5. Review Comments to the Author

Please use the space provided to explain your answers to the questions above. You may also include additional comments for the author, including concerns about dual publication, research ethics, or publication ethics. (Please upload your review as an attachment if it exceeds 20,000 characters)

**Reviewer #1**: This is a significant study on a large data base (longitudinal). While the study has a lot of merits, I would like to invite the authors to clarify the following issues through revision:
1. What are the strengths of exploring only ‘the cognitive and behavioral learning strategies from Pintrich’s SRL model (not with motivation)?

2. What is the KELS database about? I think that the database needs to be described when it was mentioned for the first time in the main text.
3. What do the authors mean by individual differences in self-regulation？
4. The writing needs to present a much more compelling argument for the research questions to be addressed in the inquiry. It looks that these questions have already been examined and answered in previous research.
5. Please indicate how each research question was addressed by the results in the findings’ section.
6. ‘Twenty-first century learners need to have learning opportunities to activate various…’. Does this mean that learners in other centuries do not need?
7. I think that the findings should add to what we know about self-regulation and academic achievements. Not sure if the study adds to our understanding of self regulation and academic achievements. The discussion needs some explicit comparison between the study’s findings and those of previous research. Apart from confirming those of previous research, what else did the study achieve?

We appreciate your positive evaluation comments and additional questions to develop the manuscript. Here, we provided more details about the collected data structures and general information of this study that we cannot include in the manuscript due to the length limitation.

A1-2. The KELS database is the collected national educational data by the South Korean Educational Department. The government collected this large-scale scale student data to collect demographic information; the main goal of the national data collection is for checking the students’ academic achievements and evaluating the current national. The dataset was shared for permitted researchers only for research with a non-interest purpose. The original dataset focused on explaining students' perception of self-regulated learning strategies, which were basis on the part of Pintrich’s SRL model from the cognitive and behavioral learning strategies only. The original dataset collected the students’ learning motivation data, but the basis was on a different theoretical model (i.e., self-determination theory by Ryan & Deci (2000). Thus, considering the current data structure, we focused on the students’ learning strategies and self-regulation topic on Pintrich’s theoretical model. In addition, our team prepares a separate study under the SDT theory focusing on the motivation and emotion topics. Reflecting on reviewer’s comments, we added additional information about KELS in the introduction to help readers better understand this secondary data analysis study.

A3-4. A self-reported survey measured the self-regulated learning strategies, and students answered how frequently they use the specific learning strategy related to the self-regulation dimension. Thus, it could explain how students use self-regulated learning strategies as active learners. The unique point of this study is focusing on young learners’ self-regulation development because we had a basic assumption that students' self-regulation could be developed longitudinally until becoming adults. Because elementary students may show different self-regulation patterns than college or adult learners, we intended to explore how successful 6th-grade learners frequently use some strategies as active learners. This study has an approach to developmental perspectives on self-regulation. We highlighted the findings focused on the previous lit-review that collected data from high school and college students or the majority in the Western population. In South Korea, young students’ self-regulated learning was not fully explained with a large sample at the national dataset level; thus, we believe the added value of this study would provide insights into how students’ self-regulated learning strategies can multidimensional perspectives on young students growing in the Eastern culture.

A5. We appreciate the suggestion. In the revision, we matched the research questions and preliminary discussions; please see the revised parts in our discussion section.

A6. Thanks for pointing this out. We revised the discussion section's first paragraph and provided a short summary of our results. We agree that procedural and conditional knowledge would be getting important for all school-aged children across all the time. To avoid using unclear expressions, we deleted the previous sentence that a reviewer pointed out.

A7. Regarding the above answers, the value and progress of this study can be explained in two ways: the first is to explore the development aspect of the self-regulated learning strategies of sixth-grade students compared to previous studies, which were mostly conducted with adult learners. The self-regulated strategy should not be equally developed; we thought some strategies might develop later than others strategies. Even students with high achievement may not frequently use some strategies compared to successful adult learners. This should be explained with a developmental approach, so this study may contribute to providing suggestions for elementary teachers regarding helpful learning guides for learners at that age level. In addition, the second is to shed light on the differences in self-regulated strategy development of school-aged children who are growing up in Asian culture. In the discussion, we reviewed how self-regulation strategies in school-age children may differ from successful adult learners and further highlighted the different patterns seen by learners in different cultures.

**Reviewer #2**: This is a cross-sectional study. For the current report, authors used the Korean ELS Study and reported if the association between some cognitive elements and kids scores vary based on the context they are present. The context on this study is mainly private vs public school. Although the sample size is large, and there were opportunities for a great paper, authors did not take the best advantage of such a great data. Here are my main concerns about this paper:

1- The main reason I would be interested in this paper would be the groups differences not the main effects. This includes main effects of school. Of course, we know that higher cognitive capacity is good. Of course, we know that private schools are more successful than public schools. These are questions that have been answered decades ago. What would be relevant and interesting here are not those questions but whether these processes vary based on the context. Unfortunately, this is only superficially touched, and there is no good conversation / discussion on this topic. For example, the conclusion is full of general points irrelevant to school context.

Thank you for your helpful feedback. After reviewing the feedback comments, we decided to revise the title to show the direction of this study more clearly. This study explores the development of self-regulated learning skills of young students at the 6th-grade level and the difference in academic achievement by using two-level HLM analysis. We further explored whether there are differences according to school groups by reflecting on clustering issues at the second level. As you point out, the value of this study is a large enough sample for using the multi-level analysis. After the analyses, we found that clustering considerations at level 2 provide statistically better understandings, but also these clustering-based differences are around 14-19% on achievement (see the result section on ICC level), and thus the more significant 80% differences are explained at level 1 individuals.

To be specific, in the HLM, we added the individual psychological variables as the level-1 predictors. However, all the predictors of level-2 are the school grouping variables (Type, Urban, and Suburban); the level-2 predictors' slopes tested the group difference. The coefficients for school location and school type are testing the group differences in the random slopes (we newly provided the appendix, including a two-level HLM model structure).

We understand the previous title makes readers have an expectation of school differences; thus, readers may have questions about the study’s primary findings and interpretation of the study. This would be a mismatching title issue and less tightly linked to the real benefits of using 2-level hierarchical linear analyses. Thanks for sharing your opinion and concerns.

2- Regarding the structure, we need to see limitations and future directions (not the reasons of conflicting results with the literature) in such sections. We need to see the aims of the study at the end of the background/intro, not the 2nd paragraph of the intro. We need to see more specific terms in the conclusion. We wish to see the summary of the study findings in the beginning of the discussion.

I appreciate your suggestions for adjusting the structure. To make a better structure, we entirely adjusted the discussion section and a part of the intro. For example, we moved down the paragraph of the research gap (intro) to the end of the background section. We revised this paragraph to be linked to the next sections. Also, we rewrote the first paragraph of the discussion section to help readers see the summary of the study findings.

3- Regarding the measures, reliability is given, but the range of the score, the thresholds used, and the meaning of the scores are not given. There are no indications of the validity either.

Thanks for sharing your concerns about the measurement issues. We provided additional information on measurements and the original database. Also, Table 2 shows the descriptive results of M, SD, and ranges of measured variables.

4- Regarding the tables, in any section that presents data by group, we need to see p values. We also need to see the name of the statistical test below that table. Currently, only regression tables give p values.

Thanks for sharing your concerns. The results of the 2nd-level slope variable in the table show the statistical differences among the groups. T-test was conducted for each coefficient. In the HLM, we added the individual psychological variables as the level-1 predictors. However, all the predictors of level-2 are the school grouping variables (Type, Urban, and Suburban); the level-2 predictors' slopes tested the group difference. The coefficients for school location and school type are testing the group differences in the random slopes (we newly provided the appendix including a two-level HLM model structure). Non-significant slope show there were no statistical differences between the different groups. HLM model already included relevant information about the various predictors (individual level and school grouping levels), so to make a parsimonious analytic model, we explored all individual factors and school grouping factors in level 1, additionally added the school grouping variables into level 2 focusing on the significant SRL variables. We got a consult about those complex analytic procedures from a professor in the statistic and measurement program in the stage of the analytic model development. Rather than reporting the ANOVA results among the group, we decided to report results from the dummy variable in the HLM model because this was the primary data analysis model for this large-scale dataset considering the clustering method.

**Reviewer #3**: Abstract
The abstract provides a clear overview of the study’s aim to explain the relationships between self-regulated learning strategy use and academic achievement of 6th-grade students in South Korea. It also mentions the use of a large existing database, the Korean Educational Longitudinal Study (KELS), which allows for a detailed analysis of the relationship between self-regulated learning strategy use and academic achievement. The use of a large dataset is a strength as it allows for a more representative sample and increases the generalizability of the findings.
The abstract also mentions the use of 2-level hierarchical linear models (HLM), which is an appropriate statistical method for analyzing data with a nested structure, such as students within schools. Additionally, the abstract highlights the consideration of environmental factors such as school type and location, which is a valuable aspect of the study as it allows for a more comprehensive understanding of the relationship between self-regulated learning strategy use and academic achievement.
The abstract also presents the study’s key findings, including that metacognition and effort regulation positively predicted literacy and math achievement and that average literacy and math achievement were significantly higher in private schools than in public schools. However, the abstract could be improved by briefly explaining the study’s limitations and novelty. This is important as there are several studies on math and self-regulation. Overall, the abstract is clear and summarises the study’s aims, methods, and key findings well.

Thanks for the positive comments and for highlighting the value of this study. We are glad to hear that the study aims were delivered to you, and we highlighted the novelty in the revised abstract.

Introduction
The author presents a well-written and detailed background on the importance of self-regulation skills in learning and the lack of emphasis on teaching these skills in the K-12 curriculum. They also provide a clear overview of the conceptual frameworks and previous research on self-regulated learning (SRL) strategies in elementary students, highlighting the limitations of small sample sizes in past studies. The use of the Korean Longitudinal Educational Study (KELS) database, which offers a large and nationally representative sample, is a strength of the study. The author also thoroughly reviews the relevant literature and previous studies using the KELS database. However, it would be beneficial for the author to provide more specific details on the research question and methodology of the current study and how it will contribute to filling the gap in the literature on SRL in elementary students. The novelty should be stated in the introduction specifically, as it was not stated in the abstract.

Thanks for your positive evaluation. Following your suggestions, we additionally stated the research gap focusing on the developmental perspectives (young learners’ self-regulation development) and different learning contexts (Asian country contexts).

Literature review
The author provides a thorough and well-written literature review on the learning strategies of self-regulated learners. They clearly explain the importance of self-regulation in academic achievement and cite relevant theories and studies in the field. The use of Pintrich’s model of self-regulated learning and the organization of the strategies into cognitive and behavioural categories is a strength of the review. The author also explains the specific types of cognitive strategies, such as rehearsal and elaboration, and behavioural strategies, such as time and effort regulation, and how they relate to self-regulation and academic achievement. However, the literature review could be improved by including more recent studies and providing a more detailed synthesis of the literature to better identify any gaps or inconsistencies in the literature and how the current study aims to address them.

I appreciate your considerable comments. We revised the lit-review section. The other reviewer pointed out that the previous lit-review section is too long, so we aggregated some statements to highlight common findings from the multiple references and added some new citations on the topic.

The research problem presents a novel contribution to the literature by investigating the relationship between South Korean 6th-graders’ use of self-regulated learning (SRL) strategies and their academic achievement in literacy and math. The study is also unique in that it specifically focuses on the early developmental stage of SRL strategies in students of this age group. The use of Pintrich’s model of SRL and the inclusion of both cognitive and behavioural strategies as the features of successful self-regulation is also noteworthy. The research questions, specifically Q1 and Q2, aimed to investigate the relationship between SRL strategy use and academic achievement, while Q3 and Q4 aimed to investigate the moderating effect of school type and location on this relationship, which provides some novelty. Overall, this research problem presents a novel and valuable addition to the field as it aims to fill the gap in the literature on SRL strategies in early developmental stage students in a specific cultural context.

We appreciate so much that you highlighted the values of this study.

Method
The method presented is comprehensive and includes detailed information on the data sources, study design, and measures used in the study. The use of a national dataset, the Korean Longitudinal Educational Study (KELS), is a strength of the study as it allows for a large and nationally representative sample. The inclusion of both cognitive and behavioural strategies, as well as the use of the MSLQ developed by Pintrich, provides a solid foundation for the study. The use of 2-level HLM analyses to examine predictors and moderators at the student and school levels is also appropriate for this study. The exclusion of missing data and the final sample size is also reported. The inclusion of demographic information such as gender and school location and the use of a vertical scaling approach to adjust the academic achievement scores are also noteworthy. However, it would be beneficial for the author to provide more information on the specific statistical tests used and the criteria for missing data exclusion. Additionally, it would be beneficial to provide more information on how the sample was selected if it was a random sample or not, and if the sample is representative of the population. In addition, and more importantly, the original scale for Pintrich was a 7-point scale. Please justify the 4-point scale used.

We provided additional statements related to missing data and sampling issues. The KELS research team conducted the pilot study to check the reliability and validity issues. Because we used the collected data as the secondary data analysis study; thus, we cannot know the exact reasons for the changes. However, we assume that the KELS team might use the 4-point scale considering young kids and having a balance with other different survey collections. The original database included the mental health questions of children, and all the survey provided items with 4 or 5-point scales. The MSLQ original version was developed for college students, so in this study, the survey items were adjusted for elementary participants. Also, we declare that the sample is representative of the population, with purposeful sampling as the national data.

Results
The reported results are well presented.

Discussion
The discussion in this journal article presents a thorough analysis of the study’s results and their implications. The authors make a strong argument for the importance of SRL strategies in academic performance, particularly for young learners. They also critically examine the relationship between different types of SRL strategies and academic achievement, explaining why some strategies may have a stronger impact on performance than others. Additionally, they discuss the potential moderating effects of school location and type on this relationship.
The authors use previous studies on the topic to support their arguments and provide context for their findings. They also highlight the limitations of the study and potential areas for future research. Overall, the discussion demonstrates a sound understanding of the theoretical foundations of the study and a critical evaluation of the results.

Thanks for your positive evaluations and thoughtful comments.

Conclusion
The conclusion is clear and flows well. It highlights the importance of SRL strategy use as a key learning skill for future generations, and the study’s findings have important implications for future research and practice in K-12 education. The conclusion suggests that elementary students should have more opportunities to develop SRL skills and that schools should provide an appropriate learning experience to help students develop these skills well. The conclusion also highlights the importance of metacognitive strategies in learning and the need for teachers to help children learn to plan, monitor, and evaluate their learning processes during classroom activities. The conclusion also notes the need for educational researchers to work closely with teachers to incorporate these theoretical principles into specific subject curricula.

The conclusion also points out that teachers’ roles in guiding SRL strategies need to be better supported in school environments, including public schools and rural areas, where achievement gaps are evident. It also highlights that school location was an influential factor in mathematical achievement, suggesting that there is a particular need to support SRL strategies for rural students in math education to prepare them for future STEM education. The conclusion also suggests that promoting metacognition, task-regulation, and effort-regulation learning strategies could help young students to increase their learning efficiency. Overall, the conclusion makes a valuable contribution to the knowledge of the field by providing practical recommendations based on the study’s findings.

The study also found a notable achievement gap between public and private schools in South Korea and highlights that future research should consider students’ socioeconomic background to explain the gaps in achievement and learning strategies. This research can serve an important social justice purpose by addressing equity issues, and as such, it should be highlighted.
There are a few typo errors noted in the track changes manuscript. Please address.

We appreciate your thoughtful and positive comments. We accepted those minor tracked changes in the manuscript and double-checked the final editing issues among the authors. Thank you so much!

**Reviewer #4**: The manuscript titled “Self-regulated Learning Skills and Academic Achievement in South Korean 6th-graders Based on Varying School Environments” is an interesting study that complies with the PLOS ONE standards. It´s an interesting, well conducted work regarding the Learning Skills and Academic Achievement area of emphasis. However, it has some weaknesses that should be considered.
Overall, my main concern is:
I consider the document too long, making it difficult to read and understand its content. I believe that it would be beneficial to synthesize some parts of the article (I will mention them in the specific considerations), which would make the document more concise and, consequently, easier to read and understand. In addition, the authors must correct the entire document, regarding the bibliographic references. They used the “APA” norms, when the formatting of the journal requires “Vancouver”.

I appreciate your feedback and suggestions. We carefully read the comments and tried to develop the manuscript reflecting your suggestions. We tried to make a clear structure to deliver insightful findings to readers. Please see the tracked changes and response comments for all reviewers. We edited the manuscript following the Vancouver style.

Some specific considerations:

Introduction / Background:

Although the content of this chapter is adequate, I propose to summarize. There is too much information, which is not used later, namely in the discussion of the results. Basically, the theoretical framework has an excess of information and content (although adequate). I propose that the authors limit themselves to the essential content, which should be restricted to what is used for the discussion of the results.
On page 9 (line 13), there is a reference without date.

Thanks for your feedback. We generally cut some paragraphs (Intro and Lit-review) that broadly introduce the theoretical backgrounds to clarify the manuscript within the appropriate length. Also, we added the correct reference information on page 9. Please see these revised sections.

Study Purpose and Research Questions:
The first sentence of this chapter needs to be changed. It's very confusing.

Thanks for the suggestions. We revised the first paragraph on the Study purpose and tried to make the expressions to be clear and coherent.

Method:
In the “Data Analysis” part, an exhaustive description of the statistical methods used in all the data analysis that was carried out is required. They must describe all the statistical techniques used as well as the significance levels.

Thanks for the feedback. One of the co-authors is a statistics expert in the HLM analyses. We reviewed the method section and included additional explanations of the analytic model and procedures (See the updated manuscript and the Appendix document).

Results:

The content of this chapter should also be more abbreviated. Namely, authors should limit themselves to presenting the results and, at most, highlight some results. What happens is that, at times, the results are practically discussed, which should be referred to the discussion chapter.
I do not agree that the comparison between public and private schools (School Types) can exist and be used in this study. This observation has to do with the fact that, out of a total of 446 schools, only 12 are public (2.7% of the sample). I propose that this comparison be withdrawn.

I appreciate the suggestion of making a shorter result section focusing on the primary findings. We cut out the redundant explanations and provided the main results in this section. The relevant depth interpretations were provided only in the discussion section. The entire manuscript was shortened compared to the original submission.

This national education dataset was collected with consideration of the Korean educational context. Most South Korean elementary schools are publicly operated under government control, with less than 3% being operated by private foundations. The study sample included 6717 public and 348 private school students, with the ratio determined based on the distribution of school types in South Korea. Despite the small representation of private schools, a large sample size (n = 348) allowed for meaningful analysis and consideration of schools s an important environmental factor in students' outcomes. Expert consultation was sought, and it was decided to include school type as a variable; we also commented on this issue in the limitation section. While the original dataset did not collect information on students' economic status, it is recognized that private school students in South Korea come from middle or upper-class families. Therefore, we believe the school type may provide insightful results related to students’ family environment as the developmental perspectives of young learners.

Discussion/ Conclusion:

At the beginning of this chapter, please recall the main objective of the study and also the main findings.
Once again, the conclusion has too much information. I suggest that the authors limit themselves to drawing the conclusions arising from the analyzes carried out on their results and ignore everything else

Reflecting on the reviewer’s suggestions, we deleted the redundant expression across the lit-review, results, and discussion sections. Thus, the revised manuscript has fewer words compared to the original version. We tried to provide insightful findings that were only tightly linked to our research questions.
